# Editors’ Book Table

**Published:** 1873-12

**Authors:** 


### Editors' Book Table.

[NOTE. — All works reviewed in the columns of the CHICAGO MEDICAL JOURNAL may be found in the extensive stock of W. B. KEEN, COOKE & CO., whose catalogue of Medical Books will be sent to any address upon request.]

BOOKS RECEIVED.

*The Diseases of the Prostate, their Pathology and Treatment, comprising the Jacksonian Prize Essay for the Year* 1860. By SIR HENRY THOMPSON, F.R.C.S., Surgeon Extraordinary to H. M. the King of the Belgians, etc., etc. Fourth Edition. Philadelphia: Henry C. Lea. 1873.

The republication in America of this, the fourth, edition of Sir Henry Thompson's valuable monograph, cannot fail to be welcomed by the practitioner solicitous for the cultivation of accurate diagnosis in this much neglected field of pathology. The literature of this department of practice may have been hitherto represented by a blank. This vacuum no longer exists, and the practitioner seeking instruction in examination and treatment of prostatic disease, at this day of such frequent occurrence, will find in this little work a mass of clinical data collated with judgment, of propositions rationally sustained, and of conclusions logically deduced. The pathological parallelism between prostatic and uterine disease, deduced from anatomical homologies, so singularly ignored by the vast majority of practitioners, is clearly and satisfactorily established. Embodied in the work are valuable intructions upon catheterism and upon vesical injections. The book is a condensed summary of the state of our knowledge of the disease down to the present day, derived not only from the author's extended field of observation, but from the literature of the science. H.

*Clinical Electro-Therapeutics, Medical and Surgical.* A Hand Book for Physicians in the Treatment of Nervous and other Diseases. By ALLAN McLANE HAMILTON, M.D., Physician in charge of the New York State Hospital for Diseases of the Nervous System, etc., etc. New York: D. Appleton & Co., 549 and 551 Broadway. 1873.

One of the most intelligible books upon the subject, in the language. The subject of electro-therapeutics may be said to have entered upon a new era in its history, the era of scientific investigation and logical analysis. After having for so many years constituted the stock in trade of itinerant charlatans, it seems now about to be rescued from their hands, and destined to prove the grand resource against so many of the *opprobria medicorum* which still cumber the catalogue of nervous diseases. Every effort to bring this powerful agent, electricity, within the reach of general practitioners, and reduce its operations to limits of laws, and to determine the principles of their action, deserves commendation. This appears to have been the aim of the author of this little treatise, and he has certainly attained more nearly the accomplishment of his object than any one who has preceded him.

Without attempting to exhaust his subject, or the patience of his readers, he has given us a practical manual, which can be made useful by any tyro acquainted merely with the rudiments of the science. H.

*Hand Book of Physiology.* By WILLIAM SENHOUSE KIRKES, M.D. Edited by W. MORRANT BAKER, F.R.C.S.E., etc.; with two hundred and forty illustrations. A New American, from the Eighth enlarged English Edition. Philadelphia: Henry C. Lea. 1873.

"*Vox Populi, Vox Dei,*" says the old proverb, and proverbs, if not all fact, are at least "founded on fact." When the demand for a book has been so great as to rapidly exhaust eight editions, criticism is superfluous. The profession at large has already recorded in the most practical form its favorable criticism upon the work, and it only remains for us to say to the small remainder of our readers who have not yet possessed themselves of the book, that we most heartily endorse the public verdict, that it is the most useful handbook of physiology accessible to the American practitioner, and feel assured that when they have read it for themselves, they will thank us for calling their attention to it. H.

*The Principles and Practice of Medical Jurisprudence.* By ALFRED SWAYNE TAYLOR, M.D., F.R.S., Fellow of the Royal College of Physicians, Lecturer on Medical Jurisprudence in Guy's Hospital. Second Edition. 2 vols. Philadelphia: Henry C. Lea. 1873.

In the November number of the JOURNAL we took occasion to call the attention of the profession to the Manual of Medical Jurisprudence by the same author, and published by the same house, as the truly great work now before us. We have little to add in commendation of this, to what has been already said of the Manual, beyond putting our expressions relative to that in superlatives. The Manual contains enough and more than enough to supply the professional needs of the general practitioner; but the complete work now before us will prove to the expert a perfect mine of wealth, in which he may delve most laboriously without fear of exhausting its treasures. The publisher, in giving this work to the profession so soon after the publication of the Manual, has given practical expression to his confidence in the intellectual appreciation of American physicians and jurists, which they should not be slow to justify. The subject matter of this work seems to be brought down literally to the present day. H.

*Treatise on the Diseases of the Eye, including the Anatomy of the Organ.* By CARL STELLWAG, (von Carion), Professor of Ophthalmology in the Imperial Royal University of Vienna. Translated from the fourth German edition, by D. B. ST. JOHN ROOSA, M.D., Clinical Professor of Diseases of the Eye and Ear, in the University of the City of New York, etc., etc.; CHAS. S. BULL, M.D., formerly Assistant Surgeon to the Manhattan Eye and Ear Hospital, etc., etc.; CHAS. E. HACKLEY, M.D., Clinical Professor of Diseases of the Eye and Ear, etc., etc. Fourth Revised and Enlarged Edition. Illustrated by wood engravings and chromo-lithographs. New York: William Wood & Co. 1873.

The world-wide reputation of the author of this work, together with the merited claims to distinction in the specialty of which it treats, enjoyed by its American editors, should earn for it the same welcome reception accorded to the first American edition. One must use much diligence indeed to find occasion for adverse criticism, notwithstanding the magnitude of the work, and the encyclopædic scope of its contents. The science of ophthalmology seems to be exhausted down to the present day to fill its pages; and the propositions advanced in the work, whether of diagnosis, pathology or therapeutics, appear to be well sustained by authorities, which are quoted, not generally, but specifically and distinctly. The work, as a whole, constitutes a most valuable addition to the library of American practitioners, few of whom are sufficiently versed in the subjects of which it treats, to enable them to dispense with the aid of an expert, even in cases involving some of the simplest forms of disease of the eyes. H.

*A Practical Treatise on Diseases of the Ear, including the Anatomy of the Organ.* By D. B. St. John Roosa, M.A., M.D., Professor of Diseases of the Eye and Ear in the University of the City of New York, etc., etc. Illustrated by wood engravings and chromo-lithographs. New York: William Wood & Company. 1873.

The author of the work before us may be truthfully called one of the pioneers of the science and art of otology in America, for it was through his translation of Von Troltsch on the Ear, published about ten years ago, that the attention of the profession in general may be said to have been first attracted to this important class of diseases. The plan of the work is very comprehensive, and the details admirably extended. Beginning with a sketch of the history of otology from the very earliest ages down to the present day, the author proceeds to an epitome of the bibliography of the science, and next, to an admirable dissertation on the anatomy of the external portion of the organ, which is illustrated by a series of the best wood cuts with which we have yet been favored. The instructions for the examination of patients are well drawn and clear, and are followed by descriptions of the diseases of this portion of the ear. The middle and internal ear are considered in their order in a similar manner, the whole concluding with some very instructive remarks on deaf-muteism and on the use of ear trumpets. The chromo-lithographic illustrations of the various forms of tympanic diseases are the best we have ever seen. There is scarcely a practitioner who is not often consulted in regard to aural diseases about which he knows nothing whatever, his therapeutical range being limited to local applications to the meatus externus. The careful study of this work will enable him to treat successfully many of the simpler forms of aural diseases without the aid of the generally inaccessible expert or the omnipresent charlatan. H.

*A Treatise on the Diseases of the Eye.* By J. SOELBERG WELLS, F.R.C.S., Doctor of Medicine of the University of Edinburgh, etc., etc., etc. Second American, from the third English Edition, with additions; illustrated with two hundred and forty-eight engravings on wood, and six colored plates, etc., etc. Philadelphia: Henry C. Lea. 1873.

Coincidently with the appearance of the great work of Stellwag Von Carion, bearing the "imprimatur" of W. Wood & Co., of New York, appears this splendid addition to ophthalmological literature from the press of Henry C. Lea, of Philadelphia.

Had we to choose between the two, we fear that we should find ourselves in the predicament of the fabled ass between the bundles of hay, and should certainly take both. Had we seen but one, we should certainly have considered it the most valuable compendium of ophthalmology in the English language, but with both before us we say most heartily to our readers, buy both, read both, and you will have learned more of the diseases of the eye than you ever knew before. H.

*Transactions of the American Medical Association.* Vol. 24. 1873.

The present volume of the "Transactions" is about up to the average, possessing, however, some points of unusual interest, in the statistical tables of Medical Societies and of Hospitals in the United States, and in its Necrological Report. The "address" of the President has been so thoroughly criticized that we will not attempt it, satisfied that we could not do justice to the subject; it certainly has the merit of originality, the style being imitated from no classic model, either ancient or modern, with which we are acquainted. In fact, "none but itself could be its parallel."

The instructions to the publishing committee concerning the disclaimer of responsibility for the views and opinions expressed in the various papers comprising its Transactions, and published under its authority, were wise and prudent in the highest degree.

Would it not be well to appoint, at the next meeting, a committee to compile and publish, in cheap duodecimo, a detailed history of what the American Medical Association has accomplished for the profession or for science? H.

